# From Discovery to Verification: A Thirty-Three-Year Analysis of Research Design in Three Leading General Medical Journals, 1992–2024

**DOI:** 10.14740/jocmr6706

**Published:** 2026-09-26

**Authors:** Zhe Chen, Rei Gou

**Affiliations:** a Department of Therapeutic Radiology, Faculty of Medicine, University of Yamanashi, 1110 Shimokato, Chuo, Yamanashi 409-3898, Japan; b Department of Obstetrics and Gynecology, Yamanashi Red Cross Hospital, 6663-1 Funatsu, Fujikawaguchiko, Minamitsuru, Yamanashi 401-0301, Japan

**Keywords:** Meta-research, Meta-epidemiology, Clinical research, Study design, Randomized controlled trials, Evidence-based medicine

## Abstract

**Background:**

Evidence-based medicine (EBM) has reorganized clinical research around a hierarchy favoring confirmatory designs over hypothesis-generating ones. Earlier analyses rested on single-year snapshots ending in 2008, so whether the shift continued, and at what rate, across the EBM era is unknown. We characterized 33-year trends in design profile and collaboration scale in three leading general medical journals.

**Methods:**

We conducted a repeated cross-sectional analysis of PubMed publication-type indexing for the New England Journal of Medicine (NEJM), The Lancet, and JAMA, 1992 to 2024, with numerator and denominator both restricted to the Journal Article publication type. We measured the annual proportion of journal articles tagged as Phase III trials (from 1993, when that tag was introduced), multicenter studies, randomized controlled trials and case reports, and authors per randomized trial. Trends were estimated by binomial generalized linear models (odds ratio (OR) per decade). The Phase III tag was validated against 132 articles.

**Results:**

The pooled share of Phase III trials rose from 0.15% in 1993 to 5.67% in 2024 (OR 2.79 per decade, 95% confidence interval (CI) 2.59 to 3.00; P < 0.001), and multicenter studies from 4.3% to 12.6% (OR 1.25; P < 0.001). The randomized-trial share rose more modestly, from 11.4% to 13.8% (OR 1.10; P < 0.001). Case reports fell in The Lancet (7.3% to 3.8%; OR 0.78) and JAMA (7.3% to 1.3%; OR 0.63), but not in NEJM (23.0% to 25.7%; OR 0.97; P = 0.08), where stability is accounted for by two standing features, the weekly Case Records and a single-page clinical image series begun in 1993. Case reports of three or more pages fell from 11.3% to 4.2% in NEJM (OR 0.71). Median authors per randomized trial rose from 5 to 10 in 1994–1998 to 19 to 23 in 2020–2024.

**Conclusions:**

The leading general medical journals increasingly published large, multi-author, late-phase trials, while the substantive case report contracted in all three. The case report changed in kind, from a vehicle for novel observation to short pedagogical content carrying the same index tag. These trends are consistent with a structural shift from discovery toward verification, with implications for how new hypotheses enter the evidence base.

## Introduction

Since its formal articulation three decades ago, evidence-based medicine (EBM) has reorganized clinical research around a hierarchy of evidence that privileges the large randomized controlled trial (RCT) and the systematic review over hypothesis-generating designs such as the case report, the mechanistic study, and the early-phase observation [1]. The gains have been substantial: lower error rates, greater reproducibility, and reduced bias. Yet from early in the movement, and more pointedly in the decade since Greenhalgh and colleagues described EBM as a movement in crisis [2], observers have questioned whether the same evidential standards may also constrain innovation by rewarding the confirmation of existing hypotheses more than the generation of new ones.

This concern rests on an epistemic asymmetry. Many genuinely novel ideas initially enter the literature through hypothesis-generating observations or other forms of lower-level evidence: the association between *Helicobacter pylori* and peptic ulcer disease began as an observation by two clinicians that would rank near the base of any modern evidence pyramid [3]. If leading journals increasingly reserve their pages for high-level confirmatory evidence, the venues that most shape practice may under-represent the kinds of studies from which paradigm shifts have historically originated.

Two features make these leading general journals the natural place to look for such an effect. They are read disproportionately by clinicians, guideline writers, and trainees, so their content shapes what practitioners of EBM actually encounter and appraise. And, unlike the broader literature, they accept only a small, curated fraction of submitted work, which makes editorial preferences about which designs merit their limited space unusually visible.

That this shift has occurred is not itself a new observation. Examining JAMA, The Lancet, and NEJM in 1971, 1981, and 1991, McDermott and colleagues found that clinical trials nearly doubled as a share of original research, from 17% to 35%, while case series fell from 30% to 4%, randomized trials rose from 31% to 76% of all trials, and multicenter studies rose from 10% to 39% [4]. Comparing 1988, 1998, and 2008 in the same three journals, Ivanov and colleagues found that prospective randomized trials rose from 22% to 46% of original articles while non-randomized single-center trials fell from 47% to 10% [5]. The interpretation we examine here, that the emphasis on EBM has devalued the hypothesis-generating case report, has likewise been argued before [6]. What has not been established is whether the shift continued, and at what rate, across the whole EBM era. The existing studies rest on three or four single-year snapshots, the most recent of which is 2008, and none measures either the later-phase composition of the trials involved or the scale of the collaborations producing them. Twelve years after the crisis essay, we therefore set out to replicate these findings on a continuous annual series and to extend them in three ways: by carrying the series without interruption from 1992 to 2024, by separating confirmatory late-phase work from randomization as such, and by adding collaboration scale through a full census of authorship on randomized trials.

## Materials and Methods

### Data source and journal selection

We analyzed records indexed in PubMed (US National Library of Medicine, NLM) [7] for three leading general medical journals: the New England Journal of Medicine (NEJM), The Lancet, and JAMA (formerly the Journal of the American Medical Association). These were selected as leading general medical journals that set the therapeutic evidence agenda. The BMJ was not included because its research profile differs from the other three; notably, it does not publish standard case reports, redirecting them to a separate title.

### Search method and indicators

We did not search titles or abstracts for free text. Instead we counted how NLM indexers had already classified each article, using three PubMed field tags: the journal was identified by the title-abbreviation field (the [ta] tag), the year by the date-of-publication field (the [dp] tag), and the study design by the NLM-assigned publication-type field (the [pt] tag). For each calendar year from 1992 to 2024, we retrieved counts of records carrying the publication types Journal Article (the denominator), Randomized Controlled Trial, Clinical Trial Phase III, Multicenter Study, and Case Reports. Both numerator and denominator queries were restricted to records carrying the Journal Article publication type. This restriction matters for the earliest years, when The Lancet indexed much of its case-based content as letters, which carry no Journal Article tag; an unrestricted numerator set against a Journal Article denominator inflates the early case-report share. Phase III and multicenter studies served as verification indicators and case reports as a discovery indicator. An example full query is: “N Engl J Med”[ta] AND 2024[dp] AND “Clinical Trial, Phase III”[pt] AND “Journal Article”[pt]. All queries are reproducible and are listed in the companion dataset.

We began in 1992 because it is the year of the EBM Working Group’s manifesto and the year after the Randomized Controlled Trial publication type was introduced (1991); earlier years cannot be compared for these design indicators because the tags did not yet exist. The Clinical Trial, Phase III publication type was introduced in 1993, so the Phase III series begins in that year and no 1992 value is reported for it. Because single-year values are unstable at the start of that series, where the three journals together carried between one and six such articles a year, we also report a 3-year anchored comparison. We ended in 2024, the last complete calendar year before data extraction (July 2026); 2025 was excluded as a partial year and because publication-type indexing lags publication. One record in the validation sample carries a 2025 publication year in the current PubMed record but was captured as 2024 at extraction, reflecting the usual difference between electronic and print dates.

### Collaboration scale

As an indicator immune to any redistribution of case reports between journals, we measured the number of authors on randomized trials. Rather than sampling, we performed a full census: for each journal and each of three representative 5-year windows (1994–1998, 2008–2012, and 2020–2024), we retrieved every article tagged as an RCT and counted its named authors from the PubMed summary records, reporting the median with interquartile range, the mean, the maximum, and the number of trials. Records listing only a collective or group name, and therefore no named individual author, were excluded (118 of 4,068 records, 2.9%); these are concentrated in The Lancet in 1994–1998 (n = 63) and in The Lancet in 2020–2024 (n = 17), and because the recent exclusions are large collaborative trials, this choice makes the reported rise conservative. Because MEDLINE recorded at most 10 authors for citations dated 1984 to 1995 and at most 25 for 1996 to 1999, we examined the two cap regimes separately within the earliest window. The cap of 10 binds in 1994–1995, where 109 of 406 trials (26.8%) list exactly 10 authors and none lists more. The cap of 25 does not bind in 1996–1998, where the largest trial lists 20 authors. We therefore report medians for the full window and repeat the analysis restricted to 1996–1998.

### Validation of the Phase III publication type

Because the Phase III indicator carries our principal result and we could identify no published assessment of the accuracy of the Clinical Trial, Phase III publication type, we validated it. From the 1,485 records in the corpus carrying that tag we drew, before inspecting any record, a simple random sample of 100 (random seed fixed and reported in the companion dataset), and separately took a census of all 32 tagged records published from 1992 to 1999, a period from which the random sample contained no record. Each record was classified against its published report as a primary report of a Phase III trial, a secondary or ancillary report of one, a trial of another phase, not a trial report, or indeterminate, using the pre-specified rubric in Supplementary Material 1 (jocmr.elmerjournals.com). Two authors coded all 132 records independently and blind to each other; agreement was 96.2% (127 of 132) and Cohen’s kappa 0.708, and all five disagreements were resolved by joint reading of the full text. Kappa is lower than raw agreement because 93% of records fall into a single category, which is the expected behavior of the statistic under a skewed marginal distribution rather than evidence of poor reliability. We report the positive predictive value strictly (primary reports only) and leniently (primary plus ancillary reports), with exact binomial confidence intervals (CIs), and we repeat the Phase III trend analysis with annual counts reweighted by the period-specific predictive value. For the other three indicators, we relied on published assessments, summarized in the “Discussion”.

### Decomposition of NEJM case reports

Because the case-report share behaved differently in NEJM from the other two journals, we examined every NEJM record carrying the Case Reports tag and split it by title and pagination into the weekly Case Records feature (title matching Case Records of the Massachusetts General Hospital, weekly clinicopathological exercises, or the pattern Case 12-2024), single-page or electronic-only clinical image items, and case reports of three or more pages. The rule is a proxy for content and not a content classification: it cannot separate a long case report from a diagnostic-reasoning exercise, so the figure for substantive case reports is an upper bound on discovery-oriented content in every year. It was applied to NEJM only, because the case-report share declines in The Lancet and JAMA without it.

### Statistical analysis

Annual proportions were modelled with binomial generalized linear models using calendar year as a continuous predictor; we report the odds ratio (OR) per decade with 95% confidence intervals and the two-sided P value for trend, complemented by Spearman rank correlation between year and annual proportion. Analyses used Python 3.10.12 (statsmodels 0.14.6, scipy 1.15.3, numpy 2.2.6). Because these are complete indexed counts rather than a sample, the estimates describe the indexed corpus directly.

Institutional review board approval was not required because the study used only publicly available bibliographic metadata and involved no human participants or identifiable data; the work was conducted in compliance with the applicable institutional ethical standards and the Declaration of Helsinki.

## Results

Between 1993, the first year in which the Phase III publication type existed, and 2024, the pooled share of articles tagged as Phase III trials rose from 0.15% to 5.67% (OR 2.79 per decade, 95% CI 2.59 to 3.00; P < 0.001; Spearman ρ = 0.95). Because single-year values are unstable at the start of the series, the same comparison anchored on 3-year windows gives 0.17% in 1993–1995 against 4.44% in 2022–2024. Multicenter studies rose from 4.3% to 12.6% (OR 1.25 per decade, 95% CI 1.21 to 1.28; P < 0.001; ρ = 0.69). The randomized-trial share rose more modestly, from 11.4% to 13.8% (OR 1.10 per decade, 95% CI 1.08 to 1.13; P < 0.001; ρ = 0.73), indicating that the change was less in the number of trials than in their character, with the trials reaching these journals becoming later-phase and more geographically distributed (Figs. 1 and 2; Table 1).

**Figure 1:**
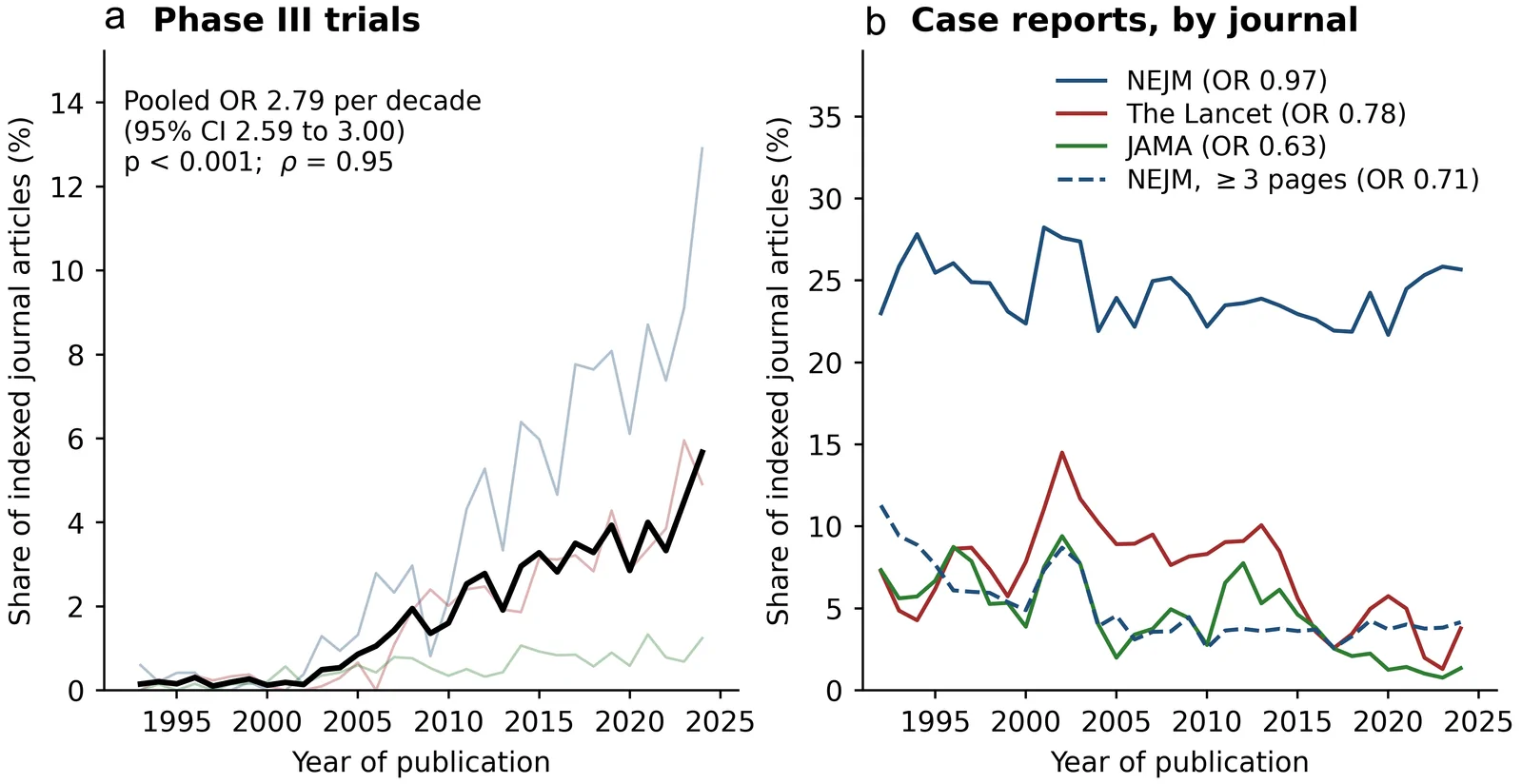
Annual share of indexed journal articles tagged as Phase III trials (a) and as case reports (b) in the New England Journal of Medicine (NEJM), The Lancet, and JAMA. Numerator and denominator are both restricted to records carrying the Journal Article publication type. Panel (a) begins in 1993, the year the Clinical Trial, Phase III publication type was introduced; faint lines show individual journals and the bold line the pooled estimate, with the pooled odds ratio (OR) per decade from a binomial generalized linear model, the two-sided P value for trend, and the Spearman rank correlation coefficient (ρ) between calendar year and annual proportion. Panel (b) shows each journal separately, because the three differ in the kind of case-based content they publish; the dashed line shows NEJM case reports of three or more pages, that is, excluding the weekly Case Records feature and single-page clinical image items. The case-report share does not fall in NEJM as a whole but falls in every journal once these standing features are separated out.

**Figure 2:**
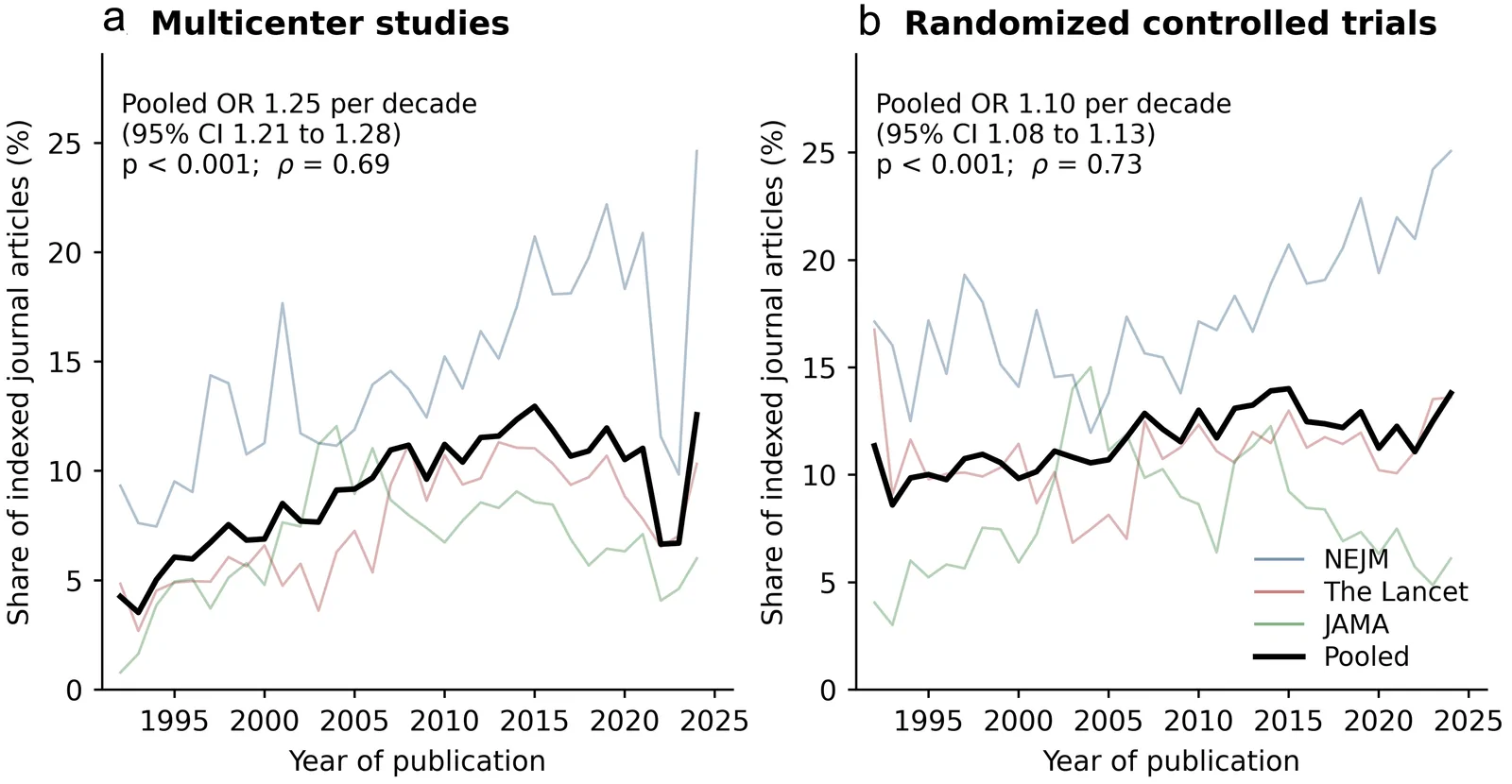
Annual share of indexed journal articles tagged as multicenter studies (a) and as randomized controlled trials (b) in the New England Journal of Medicine (NEJM), The Lancet and JAMA, 1992–2024, on the same specification as Figure 1. Faint lines show individual journals and the bold line the pooled estimate, with pooled trend estimates as in Figure 1. The pooled randomized-trial share rises modestly; the rise is marked in NEJM and weak in The Lancet and JAMA. The temporary fall in multicenter tagging in 2022–2023 is discussed in the text.

**Table 1:** Trend Statistics for Design Indicators, 1992–2024

Indicator (share of indexed journal articles)	First year	2024	OR per decade (95% CI)	P value (trend); ρ
Pooled across the three journals				
Phase III trials (from 1993)	0.15%	5.67%	2.79 (2.59–3.00)	< 0.001; 0.95
Multicenter studies	4.26%	12.57%	1.25 (1.21–1.28)	< 0.001; 0.69
Randomized trials	11.35%	13.80%	1.10 (1.08–1.13)	< 0.001; 0.73
Case reports, by journal				
NEJM, all	23.0%	25.7%	0.97 (0.94–1.00)	0.077; −0.29
NEJM, three or more pages	11.3%	4.2%	0.71 (0.66–0.76)	< 0.001; −0.67
The Lancet	7.3%	3.8%	0.78 (0.75–0.82)	< 0.001; −0.40
JAMA	7.3%	1.3%	0.63 (0.59–0.67)	< 0.001; −0.72

Data are the proportion of records carrying the Journal Article publication type in the stated calendar year; numerator and denominator are both restricted to Journal Article records. The Phase III series begins in 1993, the year that publication type was introduced, so its first-year value is for 1993; all other first-year values are for 1992. OR per decade, odds ratio per 10 calendar years, estimated from a binomial GLM; P value refers to the linear trend across years; ρ, Spearman’s rank correlation coefficient between calendar year and annual proportion. Case reports are reported by journal because the three journals differ in the kind of case-based content they publish; the NEJM three-or-more-pages row excludes the Case Records feature and single-page clinical image items, identified from title and pagination as described in the “Methods”. GLM: generalized linear model; NEJM: New England Journal of Medicine; OR: odds ratio; ρ: Spearman’s rank correlation coefficient.

In the validation sample, the Phase III tag performed well in the modern period and less well in the earliest years. Among 100 randomly sampled tagged records from 2000 to 2024, 97 were primary reports of a Phase III trial and three were secondary reports of one, giving a strict positive predictive value of 97.0% (95% CI 91.5 to 99.4) and a lenient value of 100.0% (96.4 to 100.0). None was a trial of another phase and none was a non-trial. Among all 32 tagged records from 1992 to 1999, the strict value was 78.1% (60.0 to 90.7) and the lenient value 87.5% (71.0 to 96.5); the seven records that were not primary Phase III reports comprised three letters or commentaries carrying no trial data, a prognostic modelling study, a pharmacodynamic exposure-response analysis of trial participants, an observational analysis within a trial, and an extended follow-up letter. Because accuracy is lower early and near perfect late, the uncorrected series understates rather than overstates the rise: reweighting the annual counts by the period-specific predictive value moves the estimate slightly away from the null, from OR 2.79 per decade to 2.80 (lenient) or 2.83 (strict). Full category counts, by-journal values and the coding protocol are in Supplementary Material 1 (jocmr.elmerjournals.com).

Case reports moved differently in the three journals. The share fell steeply in JAMA, from 7.3% to 1.3% (OR 0.63 per decade; ρ = −0.72), and substantially in The Lancet, from 7.3% to 3.8% (OR 0.78; ρ = −0.40). In NEJM the share did not fall (23.0% to 25.7%; OR 0.97; P = 0.08). Examining every NEJM record carrying the tag shows why. Two standing features account for the stability: the weekly Case Records of the Massachusetts General Hospital, which held steady near 40 items a year throughout, and a single-page clinical image series introduced in 1993, which grew from one item that year to about 108 a year and by 2024 supplied 16.0% of NEJM journal articles on its own. Separating these out, case reports of three or more pages fell from 52 items and 11.3% of journal articles in 1992 to 28 items and 4.2% in 2024 (OR 0.71 per decade, 95% CI 0.66 to 0.76; P < 0.001; ρ = −0.67), a slope lying between those of The Lancet and JAMA. The explicit Brief Report label, carried by 15 to 28 NEJM items a year in the early 1990s, does not appear in its titles after 1996.

The rise in confirmatory scale was not confined to the Phase III label, and the pooled randomized-trial figure conceals a changing composition: the randomized-trial share rose markedly in NEJM, from 17.1% to 25.1% (OR 1.20 per decade), and drifted upward more weakly in The Lancet (OR 1.08) and JAMA (OR 1.03, 95% CI 0.98 to 1.07). Across the indicators the common thread was not that trials became more numerous but that the trials reaching these journals became larger, later-phase and more geographically distributed. For descriptive comparison, the ratio of case reports to Phase III trials fell from about 60 to 1 in the 1994–1998 window to about 2 to 1 in 2020–2024; because case-report counts were roughly stable in absolute terms, this ratio is driven by its denominator and restates the Phase III finding rather than corroborating it independently.

Author numbers rose in parallel (Table 2). Across a full census of every randomized trial in each window, the median number of authors roughly doubled to tripled: from 10, 5, and 7 (NEJM, The Lancet, JAMA) in 1994–1998 to 23, 21, and 19 in 2020–2024. Restricting the earliest window to 1996–1998, the years in which the MEDLINE author cap could not have truncated any record, reproduces the same three medians exactly (10, 5, and 7), so the comparison is not an artifact of the cap. Means rose more steeply than medians in every journal, reflecting a lengthening right tail: the largest single trials in 2020–2024 listed 178 authors in NEJM, 82 in The Lancet, and 1,064 in one JAMA collaboration, against maxima of 20, 19, and 19 respectively in the mid-1990s.

**Table 2:** Number of Authors per RCT, by Journal and Time Window (Full Census)

Journal	Window	Trials	Median (IQR)	Mean	Maximum
NEJM	1994–1998	375	10 (6 to 11)	8.6	20
	2008–2012	584	15 (10 to 21)	17.1	141
	2020–2024	772	23 (16 to 33)	27.0	178
The Lancet	1994–1998	510	5 (4 to 8)	6.0	19
	2008–2012	449	12 (9 to 18)	14.3	266
	2020–2024	465	21 (15 to 29)	23.0	82
JAMA	1994–1998	194	7 (5 to 10)	7.4	19
	2008–2012	256	11 (8 to 17)	13.3	47
	2020–2024	345	19 (13 to 28)	26.7	1,064

Based on a full census of all RCTs indexed for each journal and window. Records listing only a collective or group name were excluded (118 of 4,068, 2.9%). MEDLINE recorded at most 10 authors for citations dated 1984 to 1995 and at most 25 for 1996 to 1999; within the earliest window the cap of 10 binds in 1994–1995 (109 of 406 trials list exactly 10 authors, none more) whereas the cap of 25 does not bind in 1996–1998 (largest trial 20 authors). Medians for 1996–1998 alone are identical to those for the full window (10, 5 and 7). Means and maxima for 1994–1998 are lower bounds and should not be compared across windows; the mean for NEJM in that window falls below its median for this reason. IQR: interquartile range; MEDLINE: US National Library of Medicine bibliographic database; NEJM: New England Journal of Medicine; RCT: randomized controlled trial.

## Discussion

In three of the most influential general medical journals, the design profile of published research changed substantially and consistently over three decades: confirmatory, large-scale designs such as Phase III and multicenter trials became several-fold more common, the typical trial grew from the work of a handful of investigators to a large multi-author consortium, and the hypothesis-generating case report contracted sharply. Taken together these convergent, independently measured trends describe a shift in what leading journals publish, from discovery toward verification. This extends the argument that Greenhalgh and colleagues [2] advanced 12 years ago: where that essay diagnosed problems of evidence volume, marginal benefit, and poor fit to multimorbidity, our data document a structural correlate. These findings need not indicate that EBM is mistaken; they are consistent with a reliability–innovation trade-off. The evidence hierarchy that made medicine more reliable also privileges designs that test established hypotheses; because novel ideas almost always debut as low-level evidence, a system optimized for confirmation may under-select for discovery at exactly the venues that most shape practice.

Two mechanisms plausibly drive the pattern. First, rising evidential expectations mean that a persuasive answer increasingly requires a large, multicenter, adequately powered trial, which in turn requires broad collaboration; hence the growth in authors and centers. The median randomized trial now carries two to three times as many authors as in the mid-1990s, and one recent collaboration listed more than a thousand, which reflects the growth of the confirmatory trial into a large, often multinational enterprise. Such scale is frequently what a definitive answer demands, but it also marks a change in the unit of knowledge production, from a small group testing a single hypothesis to a consortium delivering a precise estimate, and it helps explain why the hypothesis-generating report, by nature the work of one or a few observers, has become harder to place. Second, changes in journal scope and editorial priorities may have increasingly favored definitive confirmation over provocative observation, a shift made explicit by the redirection of case reports to dedicated titles.

Consistent with this dependence, the COVID-19 pandemic disrupted the conduct of multicenter trials in 2020 and 2021 [8], and, after the customary lag from completion to publication, the flow of multicenter-trial reports into these journals fell visibly in 2022 and 2023 (Fig. 2a) before recovering in 2024. Discovery-oriented work, which depends less on that infrastructure, appears correspondingly more robust to such shocks.

These trends may have practical relevance. A literature that more often addresses which established option is marginally better than whether current understanding of a disease is incomplete may yield diminishing returns as large, easily detected effects are exhausted, consistent with the modest effect sizes reported in contemporary Phase III oncology trials [9] and with evidence that many cancer drugs approved on surrogate endpoints show little subsequent gain in survival [10]. The trends may also be relevant to the teaching of EBM, since the journals that feature prominently in journal clubs and in the early scoping of guidelines now present a design profile weighted toward large confirmatory trials, so trainees may encounter less of the observational and mechanistic work that generates the questions trials are designed to answer. How discovery-oriented work is represented in the evidence base, and whether emerging computational methods—whose outputs are hypothesis-generating and require the same verification as other low-tier evidence [11]—alter that balance, may merit further study.

Our findings replicate and extend a line of work that runs back three decades. McDermott and colleagues [4] show that the movement we describe was already well advanced before our series begins: between 1971 and 1991 the case series had already fallen from 30% to 4% of original research in these journals, and multicenter studies had already nearly quadrupled. Ivanov and colleagues [5] carry the same direction of change to 2008. Read with the present data, the three studies describe one continuous movement across more than 50 years, from 1971 to 2024, rather than a phenomenon of the EBM era alone. This matters for interpretation. If the shift predates the 1992 manifesto by two decades, EBM is better read as having codified and accelerated a change already under way in editorial practice than as having initiated it. Our contribution is not the direction of the trend, which is established, but its continuity, its late-phase composition and its human scale: an unbroken annual series to 2024, the separation of Phase III from randomization as such, and a census of authorship showing that the median randomized trial in these journals now carries two to three times as many named authors as in the mid-1990s.

Analyzing 1990 to 2009, Wyler von Ballmoos and colleagues [12] found that in these three journals the share of randomized trials rose (20.3% to 30.9%) while cohort and case-control studies fell (34.6% to 19.2%), consistent with a move up the evidence hierarchy. They also reported an apparent rise in case reports (36.3% to 43.8%) that seems to contradict our decline, but this reflects methodological choices rather than conflicting data: their proportions are computed among a narrow set of design-classified reports (about 540 to 700 articles per year) whereas ours use all Journal Article records; their restrictive case definition captured only about a third of the items carrying the Case Reports tag; and their own counts show case reports roughly flat (195 to 246 per year) while the comparator cohort and case-control studies collapsed (186 to 108), so the apparent rise is relative to a shrinking base. Their window also closes in 2009 and misses the later fall in absolute case-report output. Read together the two studies tell a consistent story of movement up the evidence hierarchy, complementing bibliometric analyses of trial output in other literatures [13]; our contribution is to measure the shift continuously to 2024, to isolate the rise of Phase III (not merely randomized) trials, and to add collaboration scale.

Because publication-type indexing is the sole measure of design in this study, its accuracy deserves direct treatment rather than a caveat. We validated the Phase III tag against 132 source articles and found it highly accurate in the modern period, with a positive predictive value of 97.0% from 2000 to 2024, and appreciably less accurate in the 1990s, at 78.1%. That gradient matters for interpretation, because it runs against our conclusion rather than toward it: an inflated early numerator makes the observed rise a conservative estimate, and reweighting the annual counts by the period-specific predictive value moves the trend slightly further from the null.

For the randomized-trial tag we relied on published assessments, which point the same way. Wieland and colleagues found that 97% of records that appeared to describe randomized trials but carried no RCT tag did in fact report such trials, and that untagged records disproportionately reported design papers, long-term follow-up, and secondary analyses rather than main results [14]. Machine classification of MEDLINE suggests that about 5% of records tagged as randomized trials are not, and that a further 3% of randomized trials go untagged [15]; the retrieval performance of the tag has also been characterized [16]. The dominant error in these tags is therefore omission rather than false labelling, and omission has become less frequent over time. Two features of the study period work in the same direction: the Cochrane Collaboration and the NLM retagged MEDLINE records for 1994 to 2006, a window falling inside ours, which raises tag density in those years for reasons unrelated to what was published; and the Clinical Trial, Phase III publication type became available only in 1993, so that series begins in that year. Improving completeness inflates any apparent rise in trial tags, which is why our conservative reading rests on the fall in substantive case reports and on the measured accuracy of the Phase III tag rather than on the trial tags alone.

### Strengths and limitations

The study has several strengths: it uses complete indexed counts rather than a sample (including a full census of author numbers), a continuous 33-year window rather than a few snapshots, a directly measured accuracy for its principal indicator, and independent indicators of design and of collaboration scale that converge on the same conclusion, and every query is fully reproducible.

Several limitations apply, most of them properties of publication-type indexing. First, indexing remains an imperfect proxy for study design. We measured this directly for the Phase III tag and drew on published assessments for the randomized-trial tag, as set out above, but our validation estimates positive predictive value only. It does not estimate sensitivity, that is, Phase III trials published in these journals but never tagged, which would require hand searching and is the more likely source of a spurious trend, since improving completeness would inflate an apparent rise. Second, the Journal Article denominator is heterogeneous and its mix changes over time, for example a rise in Research Letters in JAMA, and records carry multiple overlapping tags, so categories do not sum to the denominator. Third, MEDLINE listed at most 10 authors for 1984 to 1995 and at most 25 for 1996 to 1999, removing the limit only from 2000, so the earliest author counts are lower bounds; the cap of 10 binds in 1994–1995 but the cap of 25 does not bind in 1996–1998, and medians restricted to those uncensored years are identical to the full-window medians, so the two- to three-fold rise is robust. Fourth, part of the decline in case reports reflects their migration to dedicated case-report journals from about 2007 to 2008, which does not affect the author metric and is itself consistent with a reallocation of space away from discovery-type content. Fifth, the decomposition of case-based content by title and pagination is a proxy for content rather than a content classification, and was applied to NEJM only. Finally, we studied three general medical journals only; specialty and basic-science venues, where much discovery is published, were outside our scope, and the discovery to verification interpretation, while supported by convergent indicators, is not itself a coded variable.

### Conclusions

Over 1992 to 2024, the leading general medical journals increasingly published large, multi-author, late-phase trials, while the substantive case report contracted in all three. The case report did not disappear from their pages so much as change in kind, from a vehicle for novel observation to short pedagogical content carrying the same index tag. These trends are consistent with a structural shift in high-impact medicine from discovery toward verification, an evolution that has improved reliability and that also has implications for how, and where, new hypotheses enter the evidence base.

## Supplementary Material

Suppl 1 Validation of the “Clinical Trial, Phase III” publication type.

## Data Availability

All data are derived from public PubMed E-utilities queries; the full annual dataset and the exact search strings are provided in the accompanying spreadsheet and can be regenerated.

## Abbreviations

CI: confidence interval
EBM: evidence-based medicine
GLM: generalized linear model
IQR: interquartile range
JAMA: Journal of the American Medical Association
NEJM: New England Journal of Medicine
NLM: National Library of Medicine
OR: odds ratio
PPV: positive predictive value
RCT: randomized controlled trial
ρ: Spearman’s rank correlation coefficient
