# Supplementary material for: From Discovery to Verification: A Thirty-Three-Year Analysis of Research Design in Three Leading General Medical Journals, 1992–2024
Source: J Clin Med Res. 2026 Sep 26;18(9):671–8. doi: 10.14740/jocmr6706 (PMC13661093; doi:10.14740/jocmr6706)
Supplement: Supplementary file 1 [file jocmr-18-09-671-s001.docx]

# Suppl1. Validation of the “Clinical Trial, Phase III” publication type

Companion to the manuscript “From discovery to verification: a 33-year analysis of research design in three leading general medical journals, 1992–2024” (jocmr6706). Part A gives the protocol, fixed before any record was inspected; Part B gives the results. The record-level codes are in the Ph3Validation sheet of the companion data workbook.

## Part A. Pre-specified protocol

## Objective

To estimate the positive predictive value (PPV) of the NLM publication type “Clinical Trial, Phase III” as applied to articles in NEJM, The Lancet and JAMA, 1992 to 2024, and to test whether that PPV differs between the earliest and the most recent years of the study window.

## Sampling frame

All 1,485 records in the study corpus carrying the Clinical Trial, Phase III publication type (NEJM 818, The Lancet 528, JAMA 139). Of these, 1,478 also carry the Journal Article tag.

## Sample

Two strata, 132 records in total, drawn before any record was inspected.

1. Main stratum: simple random sample of 100 records from the full frame (Python pandas.DataFrame.sample, random_state=20260903). All 100 carry the Journal Article tag. Distribution: NEJM 54, The Lancet 32, JAMA 14; by period, 2000 to 2009 n=8, 2010 to 2019 n=60, 2020 to 2024 n=32.
2. Supplementary stratum: a census of all 32 tagged records published from 1992 to 1999. The main stratum contains no record from this period, because only 2.2% of tagged records fall in it. This stratum exists to address the reviewer’s concern that indexing practice changed over the study window, and is analyzed separately. It is not pooled with the main stratum for the headline PPV.

The main stratum alone supports the headline estimate. With an expected PPV near 0.90, n=100 gives a 95% confidence interval of roughly plus or minus 6 percentage points, which is adequate to distinguish “the tag is broadly trustworthy” from “the tag is unreliable”.

## Reference standard

Each record is classified against its published report. Title, abstract, publication-type list and any trial-registration identifier are examined first; the full text is consulted for every record that cannot be resolved from those fields.

## Coding categories

Each record receives exactly one code.

- **1. Primary report of a phase III trial.** The report is the principal publication of a trial that is either (a) explicitly designated phase III, phase II/III or phase III/IV by the authors, the sponsor or the registry record, or (b) not explicitly labeled but substantively phase III: a randomized comparative trial in patients, with a pre-specified primary efficacy or effectiveness endpoint, intended to be definitive rather than exploratory.
- **2. Secondary or ancillary report of a phase III trial.** The underlying trial is phase III but the article is not its primary report: long-term or extended follow-up, a pre-planned or post hoc secondary or subgroup analysis, a pooled analysis of two or more phase III trials, or a mechanistic sub-study.
- **3. Trial of another phase.** Explicitly phase I, phase I/II, phase II or phase IV.
- **4. Not a trial report.** Observational study, systematic review or meta-analysis, modelling study, protocol, editorial, correspondence, or any other non-trial item.
- **5. Indeterminate.** Cannot be resolved even from the full text.

## Outcome measures

- **Strict PPV** = category 1 divided by all coded records.
- **Lenient PPV** = categories 1 and 2 divided by all coded records. This is the quantity relevant to the manuscript, because the indicator is intended to measure the volume of confirmatory phase III research reaching these journals, and a secondary report of a phase III trial is still an output of that research.

Both are reported with exact (Clopper-Pearson) 95% confidence intervals, overall and separately by journal and by period. Category 5 records are reported but excluded from the denominator, and a sensitivity analysis assigns them all to the unfavorable category.

## Coding procedure

Two authors code all 132 records independently and blind to each other. Inter- rater agreement is reported as a percentage and as Cohen’s kappa. Disagreements are resolved by discussion, and if unresolved, by a joint reading of the full text.

## What this validation does not establish

The design estimates the positive predictive value of the tag only. It does not estimate sensitivity: phase III trials published in these journals but never assigned the tag are invisible to it. Sensitivity is the more likely source of a spurious time trend, because indexing completeness improved over the study window and would inflate an apparent rise. This asymmetry is stated as a limitation, and it is the reason the manuscript’s conservative reading rests on the denominator-free ratio rather than on the phase III share alone.

## Part B. Results

Records retrieved from PubMed on 3 September 2026. Sample drawn before any record was inspected (random_state=20260903). Independent coding by ZC and RG completed 3 September 2026; the five disagreements were resolved by joint reading. Codes for both coders and the adjudicated code are provided in the accompanying coding sheet.

## Inter-rater reliability

- Agreement: **127 of 132, 96.2%** (main stratum 98 of 100; 1992 to 1999 census 29 of 32)
- Cohen’s kappa: **0.708**

Kappa reads lower than raw agreement because 93% of records fall into a single category, which is the expected behavior of kappa under a skewed marginal distribution rather than evidence of poor reliability.

All five disagreements were resolved by joint reading: two articles were agreed to be secondary reports of phase III trials (COMBI-d overall survival, PMID 26037941; EXPLORER-HCM health status, PMID 34004177), two were agreed not to be trial reports (the International Prognostic Index paper, PMID 8141877; a 1994 Lancet letter carrying no trial data, PMID 7523817), and one was agreed to be an ancillary analysis of trial participants (levofloxacin pharmacodynamics, PMID 9440662).

## Main stratum: simple random sample of 100 tagged records, 2000 to 2024

| Category | n |
| --- | --- |
| 1. Primary report of a phase III trial | 97 |
| 2. Secondary or ancillary report of a phase III trial | 3 |
| 3. Trial of another phase | 0 |
| 4. Not a trial report | 0 |
| 5. Indeterminate | 0 |

- Strict PPV (category 1): **97.0%, 95% CI 91.5 to 99.4**
- Lenient PPV (categories 1 and 2): **100.0%, 95% CI 96.4 to 100.0**

By journal, strict: NEJM 54 of 54 (100.0%), The Lancet 30 of 32 (93.8%), JAMA 13 of 14 (92.9%).

Among the 97 primary reports, 78 (80%) are explicitly designated phase III (or phase II/III, 2b/3, 3a, 3b) by the authors, the sponsor or the registry record; the remaining 19 carry no phase label and were coded on the substantive criteria in the protocol.

No record in this stratum was a trial of another phase and none was a non-trial. The three secondary reports are the COMBI-d overall-survival report, the EXPLORER-HCM health-status analysis, and a research letter reporting an exploratory hospitalisation and cost analysis of ADVANCE III (PMID 25096697). None is a misclassification of design: each reports on a trial meeting phase III criteria, but none is that trial’s primary publication.

## Supplementary stratum: census of all 32 tagged records, 1992 to 1999

| Category | n |
| --- | --- |
| 1. Primary report of a phase III trial | 25 |
| 2. Secondary or ancillary report of a phase III trial | 3 |
| 3. Trial of another phase | 0 |
| 4. Not a trial report | 4 |
| 5. Indeterminate | 0 |

- Strict PPV: **78.1%, 95% CI 60.0 to 90.7**
- Lenient PPV: **87.5%, 95% CI 71.0 to 96.5**

The seven records that are not primary phase III reports are: three letters or commentaries carrying no trial data (PMIDs 7523817, 9820292, 10359439); a prognostic modelling study built from pooled treated patients (PMID 8141877); an observational analysis of risk factors among participants in a phase III trial (PMID 10432323); an extended follow-up letter from a randomized trial (PMID 10636375); and a pharmacodynamic exposure-response analysis of trial participants (PMID 9440662).

Only 8 of the 25 primary reports in this stratum (32%) carry an explicit phase label, against 80% in the main stratum, which is consistent with a change in reporting convention rather than in indexing.

## Direction of the error and effect on the reported trend

Tag accuracy is lower in the earliest years and near perfect in the recent years. The bias therefore inflates the early phase III numerator relative to the recent one, which means the uncorrected series **understates** the rise. Reweighting the annual counts by the period-specific predictive value confirms this:

| Specification | OR per decade (95% CI) | Spearman rho |
| --- | --- | --- |
| Uncorrected (from 1993) | 2.79 (2.59 to 3.00) | 0.95 |
| Corrected by lenient PPV | 2.80 (2.60 to 3.02) | 0.95 |
| Corrected by strict PPV | 2.83 (2.63 to 3.06) | 0.96 |

Correcting for measured tag accuracy moves the estimate slightly away from the null. The headline result does not depend on the assumption that the tag is perfect.

## What this validation does not establish

The design estimates positive predictive value only. It does not estimate sensitivity: phase III trials published in these journals but never assigned the tag are invisible to it. Sensitivity is the more likely source of a spurious time trend, because indexing completeness improved over the study window and would inflate an apparent rise. This asymmetry is stated as a limitation.
